# Poroelastic properties of rocks with a comparison of theoretical estimates and typical experimental results

**DOI:** 10.1038/s41598-022-14912-5

**Published:** 2022-06-29

**Authors:** A. P. S. Selvadurai, A. P. Suvorov

**Affiliations:** 1 Department of Civil Engineering and Applied Mechanics, McGill University, Montréal, QC H3A 0C3 Canada; 2 Department of Applied Mathematics, Moscow State University of Civil Engineering (MGSU), Moscow, Russia

**Keywords:** Environmental sciences, Hydrology, Solid Earth sciences, Engineering

## Abstract

The paper develops theoretical estimates for the parameters that describe the classical theory of poroelasticity for a fluid-saturated porous medium, with a porous elastic skeleton that can exhibit imperfect grain contacts. The results for the poroelastic properties predicted from the modelling are compared with experimental results available in the literature.

## Introduction

The classical theory of poroelasticity proposed by M.A. Biot^1^ is recognized^2–10^ as a key development in the description of the continuum theory of fluid-saturated porous media. The scope of poroelasticity and related advances has found applications far beyond the originally envisaged topic of geological materials and soil mechanics. Developments in this area are too vast to cite in their entirety, and applications to diverse topics such as (1) mechanics of bone^11^, (2) hyperelastic soft tissues^12–15^ (3) poroelastic media experiencing fracture, damage, failure and irreversible processes^16–20^, (4) geologic sequestration and ground subsidence^21–27^, (5) thermo-hydro-mechanics and geoenvironmental processes^28–36^, (6) contact and inclusion problems^37–42^, and (7) in the methodologies for the estimation of the material properties of Biot poroelasticity^43–52^, are briefly documented in the cited articles.

The estimation of the poroelasticity parameters can be a challenging exercise particularly when the rock has low permeability, when saturation of an initially dry pore space requires an inordinate amount of time^49^. Also, there are no assurances that the entire pore space becomes saturated during a saturation process and the presence of unsaturated regions can lead to erroneous estimates of the poroelastic parameters. Examples where alternative approaches can be adopted, for example, for the estimation of the permeability or the Biot coefficient of low permeability rocks are discussed in^49–53^. Therefore, any theoretical procedure that can be used to estimate poroelasticity properties of rocks will enhance the applicability of the theory.

## Analytical concepts

**Figure 1:**
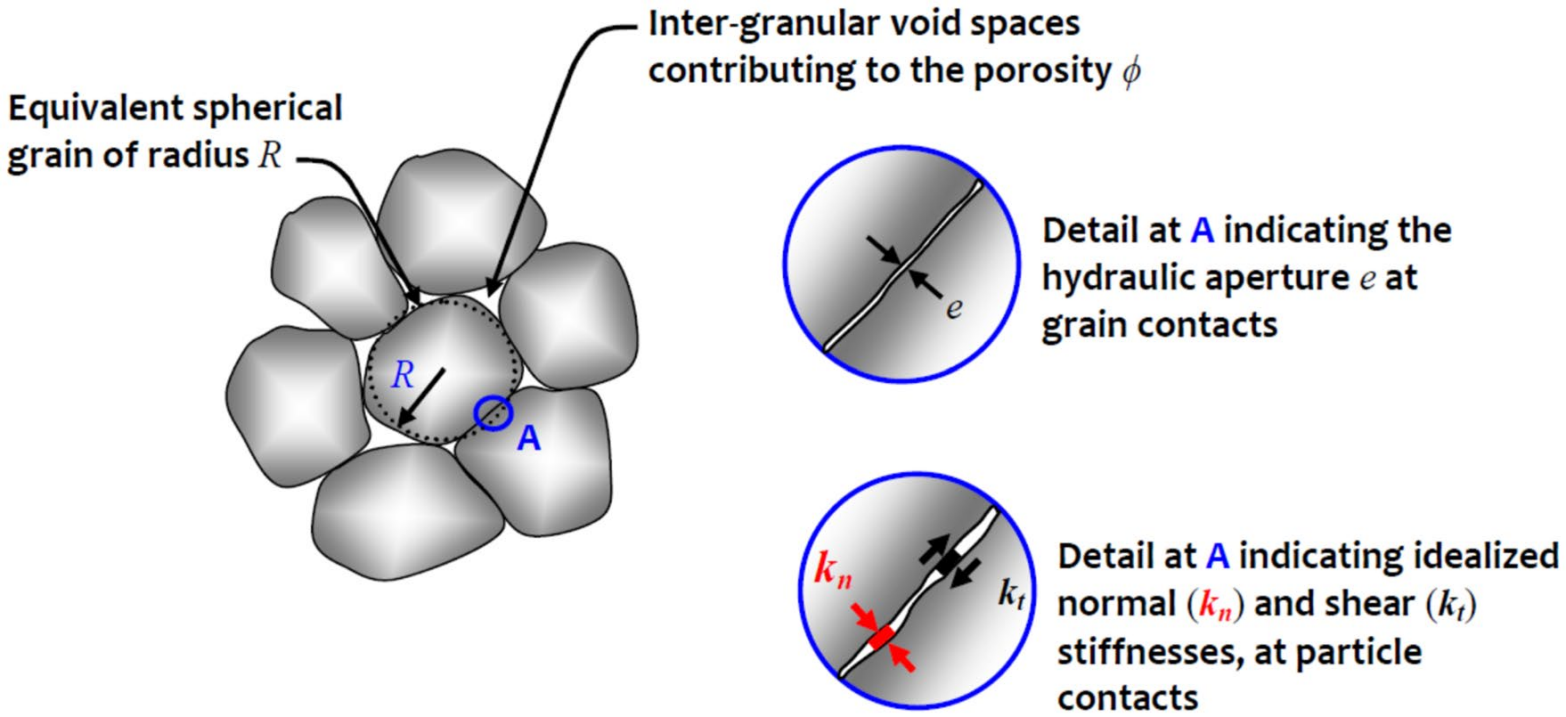
The idealized porous medium.

The classical theory of poroelasticity for an isotropic medium involves the skeletal deformability properties characterized by the skeletal or effective shear modulus ($G^{eff}$), the skeletal or effective bulk modulus ($K^{eff}$), the Biot coefficient $\alpha$ and the permeability $k$. We develop analytical estimates for these isotropic poroelastic rocks and compare the estimates with experimental estimates of these properties for various rocks such as sandstones, limestones and granites-marbles that can be obtained from^43,44^ and are presented in Tables 1 and 2, respectively. Missing properties in Table 2 are computed from available results using well known relationships applicable to isotropic elastic solids^54,55^. For example, the Biot modulus $N$ in Table 2 is computed using the relationship

$$N = \frac{{K^{eff} B}}{\alpha (1 - \alpha B)}$$

where $B$ is Skempton’s pore pressure coefficient^56^. Analytical estimates of material properties of rocks can also be obtained using the microstructural models presented in^57,58^ (see also^59,60^) . In the microstructural models, the porous rock consists of non-porous solid grains, typically spherical in shape, and the space between the grains is identified as the pore space. The porosity of rock is denoted by $\phi$. The interface between the neighboring grains is assumed imperfect and characterized by a normal contact stiffness $k_{n}$ and a tangential stiffness $k_{t}$, which can be developed by appeal to the classical studies by Mindlin^61^ and Mindlin and Deresiewicz^62^. Schematic views of the idealized concepts relating to the definitions of the granular assembly, porosity, contact stiffnesses and hydraulic aperture at grain contacts are shown in Fig. 1.

**Table 1:** Poroelastic moduli for various rocks.

Rock	$G_{eff}$	$K_{eff}$	$\nu$	$K_{u}$	$\nu_{u}$	$K_{m}$	$\alpha$	$B$	$N$	$\phi$	$k$
Ruhr sandstone	13	13	0.12	30	0.31	36	0.65	0.88	41	0.02	1.97E-19
Tennessee marble	24	40	0.25	44	0.27	50	0.19	0.51	81	0.02	9.87E-22
Charcoal granite	19	35	0.27	41	0.30	45	0.27	0.55	84	0.02	9.87E-22
Berea sandstone	6.0	8.0	0.20	16	0.33	36	0.79	0.62	12	0.19	1.87E-16
Westerly granite	15	25	0.25	42	0.34	45	0.47	0.85	75	0.01	3.94E-21
Weber sandstone	12	13	0.15	25	0.29	36	0.64	0.73	28	0.06	9.87E-19
Ohio sandstone	6.8	8.4	0.18	13	0.28	31	0.74	0.50	9.0	0.19	5.53E-18
Pecos sandstone	5.9	6.7	0.16	14	0.31	39	0.83	0.61	10	0.20	7.89E-19
Boise sandstone	4.2	4.6	0.15	8.3	0.31	42	0.85	0.50	4.7	0.26	7.89E-16

Data is taken from^44^. [The elastic moduli are expressed in GPa. Permeability $k$ is expressed in m^2^].

**Table 2:** Poroelastic moduli for various rocks.

Rock	$G^{eff}$	$K^{eff}$	$K_{m}$	$\alpha$	$B$	$N$	$\phi$
Sandstone	6.3086	8.3	34.1	0.7566	0.67	14.9063	0.19
Sandstone	5.1506	6.7	26.3	0.7452	0.85	20.8484	0.19
Sandstone	5.6262	5.7	25.4	0.7756	0.76	13.6047	0.19
Sandstone	5.4703	6.7	30.1	0.7774	0.81	18.8520	0.19
Sandstone	5.1358	5.6	28.7	0.8049	0.68	10.4514	0.19
Limestone	11.0496	20.5	70.7	0.7100	0.49	21.6953	0.13
Limestone	13.0563	22.0	74.4	0.7043	0.42	18.6304	0.13

Data is taken from^43^. [Elastic moduli are expressed in GPa].

References to these studies and other relevant expositions on contact mechanics can be found in the studies^63–68^. Applications with special reference to geomaterials are given in^69–71^. The interfaces are considered to be very thin and are assumed to have no influence on the overall porosity $\phi$. Denoting the vector normal to the interface by ${\mathbf{n}}$, the traction vector acting on the interface is denoted by ${\mathbf{T}}$, the displacement jump at the interface is denoted by $[{\mathbf{u}}]$ and the pore pressure is denoted by $p$. We can write the constitutive equations for the interface in the form

1 $$\left\{ \begin{gathered} {\mathbf{T}}{\mathbf{.n}} = k_{n} [{\mathbf{u}}{\mathbf{.n}}] - p\quad {\text{for}}\;{\text{open}}\;{\text{or}}\;{\text{permeable}}\;{\text{interfaces}} \hfill \\ {\mathbf{T}}{\mathbf{.n}} = k_{n} [{\mathbf{u}}{\mathbf{.n}}]\quad {\text{for}}\;{\text{closed}}\;{\text{or}}\;{\text{impermeable}}\;{\text{interfaces}} \hfill \\ {\mathbf{T}}_{t} = k_{t} [{\mathbf{u}}_{t} ] \hfill \\ \end{gathered} \right\}$$

where ${\mathbf{T}}_{t} = {\mathbf{T}} - ({\mathbf{T}}{\mathbf{.n}}){\mathbf{n}}$ and ${\mathbf{u}}_{t} = {\mathbf{u}} - ({\mathbf{u}}{\mathbf{.n}}){\mathbf{n}}$. Hence, if the interface is *permeable* or *open*, the fluid pressure contributes to the normal component of the traction vector, and if the interface is *impermeable* or *closed*, the fluid pressure has no influence at the interface. The *fraction of open interfaces* is denoted by $r$. Thus, $r = 0$ if all the interfaces are closed, and $r = 1$ if all the interfaces are open. Since the porosity measure utilizes only intra-granular spaces, (Fig. 1) the porosity is expected to have no influence on the interface stiffnesses $k_{n}$ and $k_{t}$.

In addition, we can consider the stress $${\mathbf{\overset{\lower0.5em\hbox{$\smash{\scriptscriptstyle\frown}$}}{T} }}$$ acting on a grain $$G_{i}$$ at the interface between the grains $$G_{i}$$ and $$G_{j}$$. We can also relate this stress to the displacement jump $$[{\mathbf{u}}]$$. Let the coefficients of proportionality in these relations for the *normal* and *tangential* components of the stress vector $${\mathbf{\overset{\lower0.5em\hbox{$\smash{\scriptscriptstyle\frown}$}}{T} }}$$ be denoted by $$K_{n}$$ and $$K_{t}$$, respectively. It is shown^57^ that

2 $$K_{n} = \frac{{k_{n} }}{2}\quad ;\quad K_{t} = \frac{{k_{t} }}{2}$$

Estimates of the elastic properties are obtained from a self-consistent method that is applicable to particulate aggregates developed in^72–74^ and summarized in^75^. The following normalized quantities are introduced

3 $$K = \frac{{K^{eff} }}{{K_{n} R}};\quad M = \frac{{G^{eff} }}{{K_{n} R}};\quad \rho = \frac{{K_{t} }}{{K_{n} }} = \frac{{k_{t} }}{{k_{n} }}$$

where $R$ is the radius of the grain. Experimental values for the grain sizes of most rocks is provided by granulometric data. In what follows, we make the assumption that the grains are rigid. In this case, the self-consistent estimate of the shear modulus can be obtained by solving the following cubic equation:

4 $$\begin{aligned} & 16M^{3} + 4\left\{ {2 + 3\phi + 2\rho (3\phi - 1)} \right\}M^{2} \\ & \quad + {\kern 1pt} \left\{ {3(3\phi - 1) + 2\rho (12\phi - 5)} \right\}M + 3\rho (2\phi - 1) = 0 \\ \end{aligned}$$

Once the normalized shear modulus $M$ is known, the self-consistent estimate of the bulk modulus can be obtained from

5 $$K = \frac{4(1 - \phi )M}{{3(2M + \phi )}}$$

From the above relationship, we have

6 $$M = \frac{2(1 - \phi )}{3}\left( \frac{M}{K} \right) - \frac{\phi }{2} = \frac{2(1 - \phi )}{3}\left( {\frac{{G^{eff} }}{{K^{eff} }}} \right) - \frac{\phi }{2}$$

Therefore, if the effective shear modulus $G^{eff}$ and the effective bulk modulus $K^{eff}$ are known from experiments, one can determine the constant $M$ from (6) and consequently estimate the ratio $\rho = k_{t} /k_{n}$ using (4): i.e.

7 $$\rho = \frac{{16M^{3} + 4(2 + 3\phi )M^{2} + 3(3\phi - 1)M}}{{8(1 - 3\phi )M^{2} + 2(5 - 12\phi )M + 3(1 - 2\phi )}}$$

It should be noted^76,77^ that self-consistent schemes are not without flaws and should be judiciously applied when extreme limiting cases are being considered.

## Numerical results for deformability behaviour

**Figure 2:**
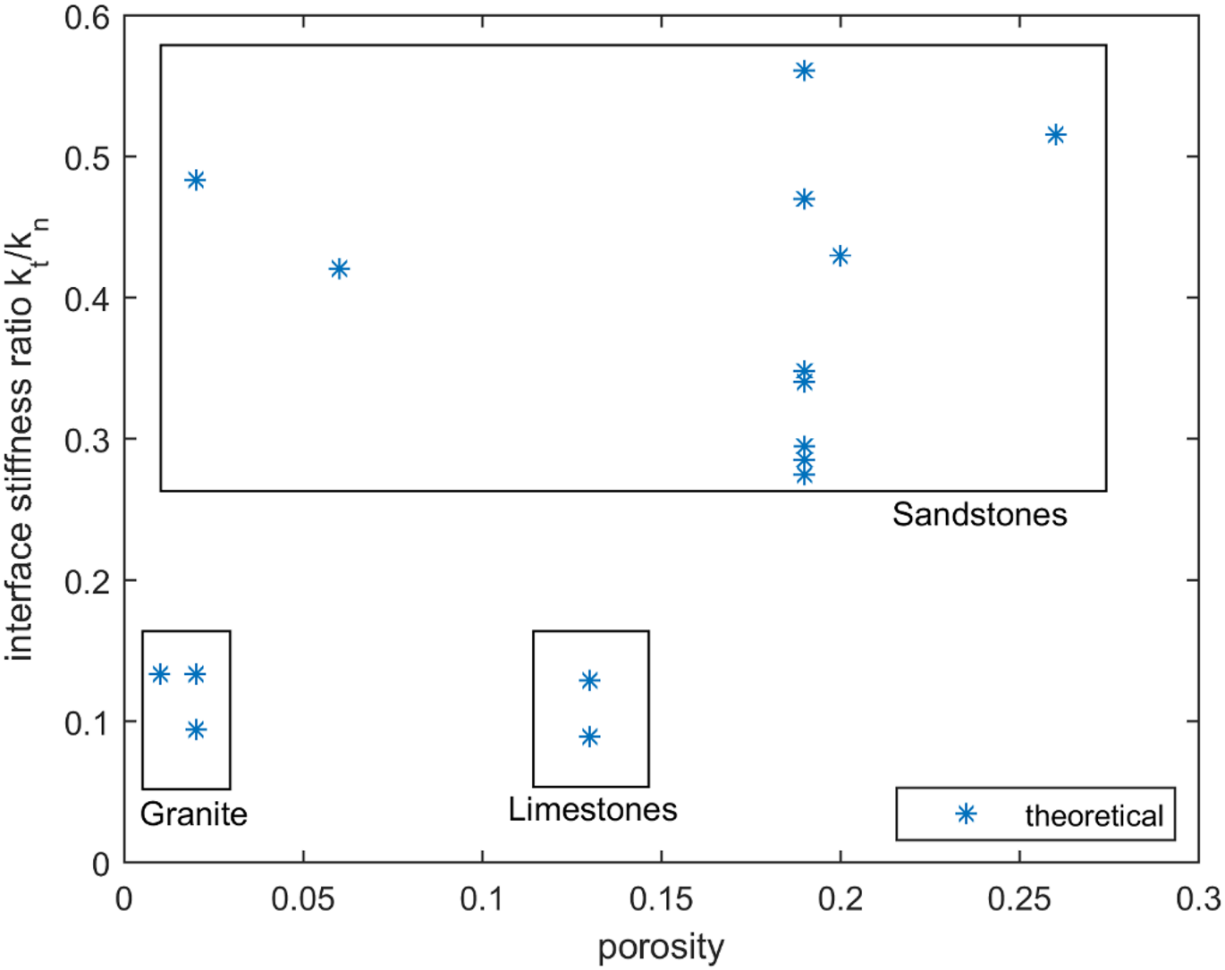
Ratio of interface stiffnesses $\rho$ versus porosity.

Figure 2 shows estimates of interface stiffness ratio $\rho = k_{t} /k_{n}$ plotted against the porosity $\phi$. The estimates are obtained from (7), based on the values of bulk and shear moduli listed in Table 1^44^ and Table 2^43^. It can be observed that the ratio $\rho$ ranges between 0.3 and 0.6 for sandstones and is approximately equal to 0.1 for limestones and granites-marbles.

**Figure 3:**
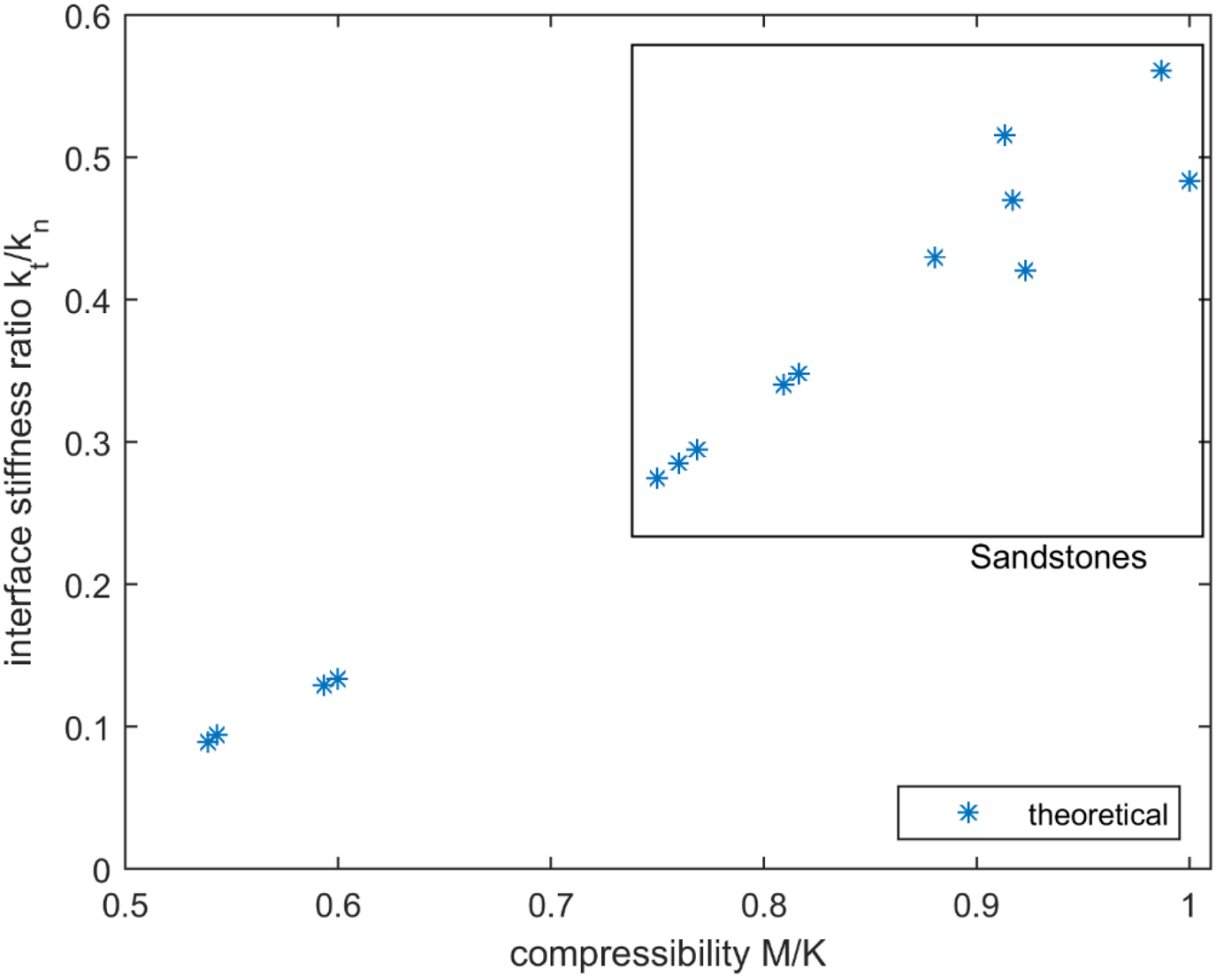
Ratio of interface stiffnesses $\rho$ versus compressibility parameter $(G^{eff} /K^{eff} ) = (M/K)$.

It is also instructive to plot the ratio $\rho$ as a function of the parameter $(G^{eff} /K^{eff} ) = (M/K)$. Figure 3 shows the ratio $\rho$ plotted against $(M/K)$. It turns out that $\rho$ is approximately a linear function of the parameter $(M/K)$.

The interfacial stiffness $K_{n} R$ can be obtained from the definition of $M$ (3), i.e.,

8 $$K_{n} R = \frac{{k_{n} R}}{2} = \frac{{G^{eff} }}{M}$$

Using (6), we also obtain the ratio of the interfacial stiffness $K_{n} R$ to the effective bulk modulus as follows:

9 $$\frac{{K_{n} R}}{{K^{eff} }} = \frac{{G^{eff} }}{{K^{eff} M}} = \frac{{G^{eff} }}{{\left( {\frac{2(1 - \phi )}{3}G^{eff} - \frac{\phi }{2}K^{eff} } \right)}}$$

**Figure 4:**
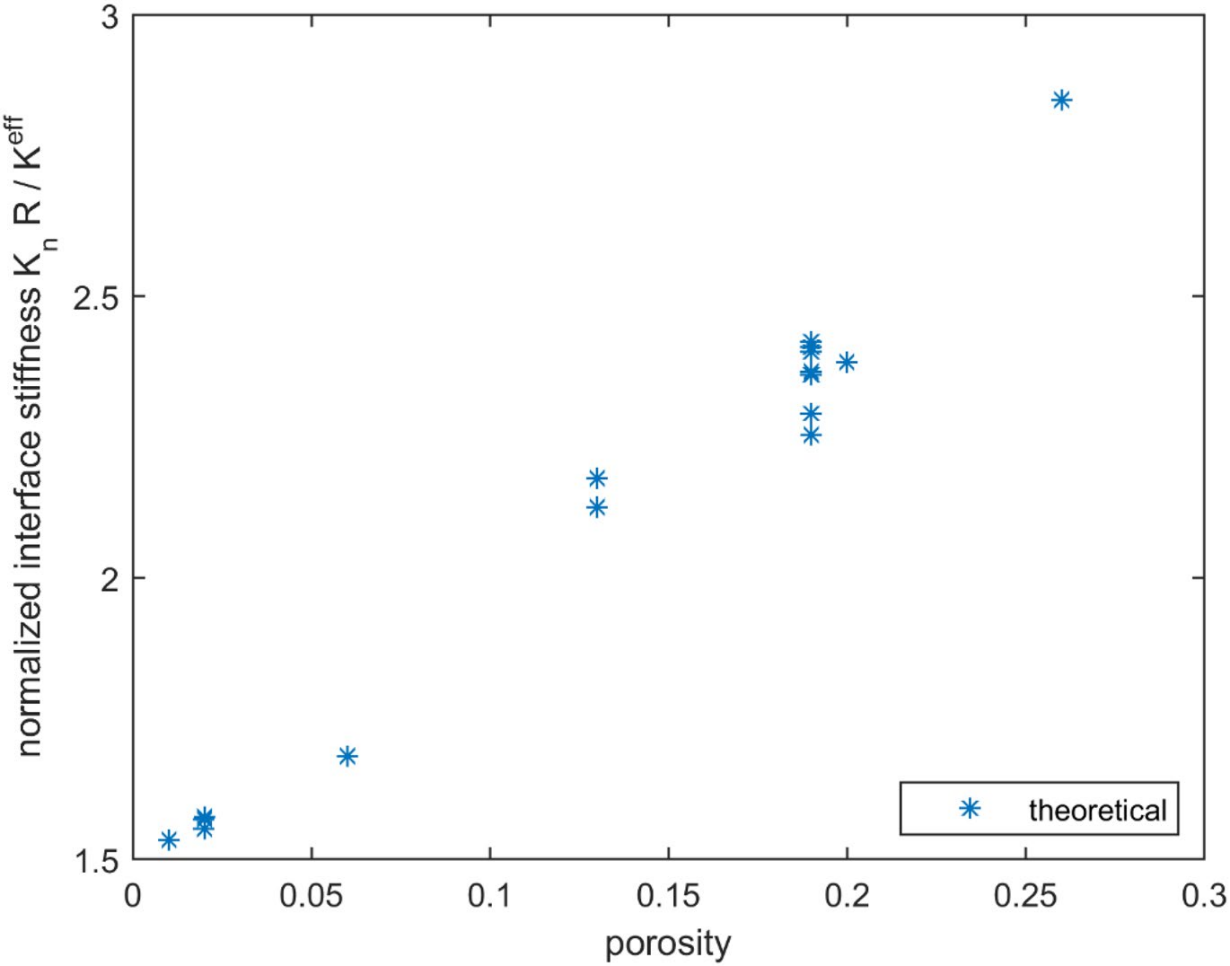
Normalized interfacial stiffness $(K_{n} R/K^{eff} )$ versus porosity.

Figure 4 shows the variation in the normalized interfacial stiffness $(K_{n} R/K^{eff} )$ with porosity $\phi$. It can be seen that this dependency can be conveniently approximated by a linear relationship.

When the grains are rigid, the estimate for the Biot coefficient $\alpha$ can be obtained in the form

10 $$\alpha = 1 - \frac{3K}{2}(1 - r)$$

where $r$ is the fraction of open or permeable interfaces, $0 \le r \le 1$. Using the expression for the constant $M$ given by (6), we can represent the Biot coefficient as

11 $$\alpha = 1 - \left\{ {1 - \phi \left( {1 + \frac{3}{4}\frac{{K^{eff} }}{{G^{eff} }}} \right)} \right\}(1 - r)$$

If the Biot coefficient is known from experimental data^10,43,44,50,51,78,79^, the fraction of open interfaces $r$ can be estimated from (11) as

12 $$r = \left\{ {\frac{{\alpha - \phi \left( {1 + \frac{3}{4}\frac{{K^{eff} }}{{M^{eff} }}} \right)}}{{1 - \phi \left( {1 + \frac{3}{4}\frac{{K^{eff} }}{{M^{eff} }}} \right)}}} \right\}$$

**Figure 5:**
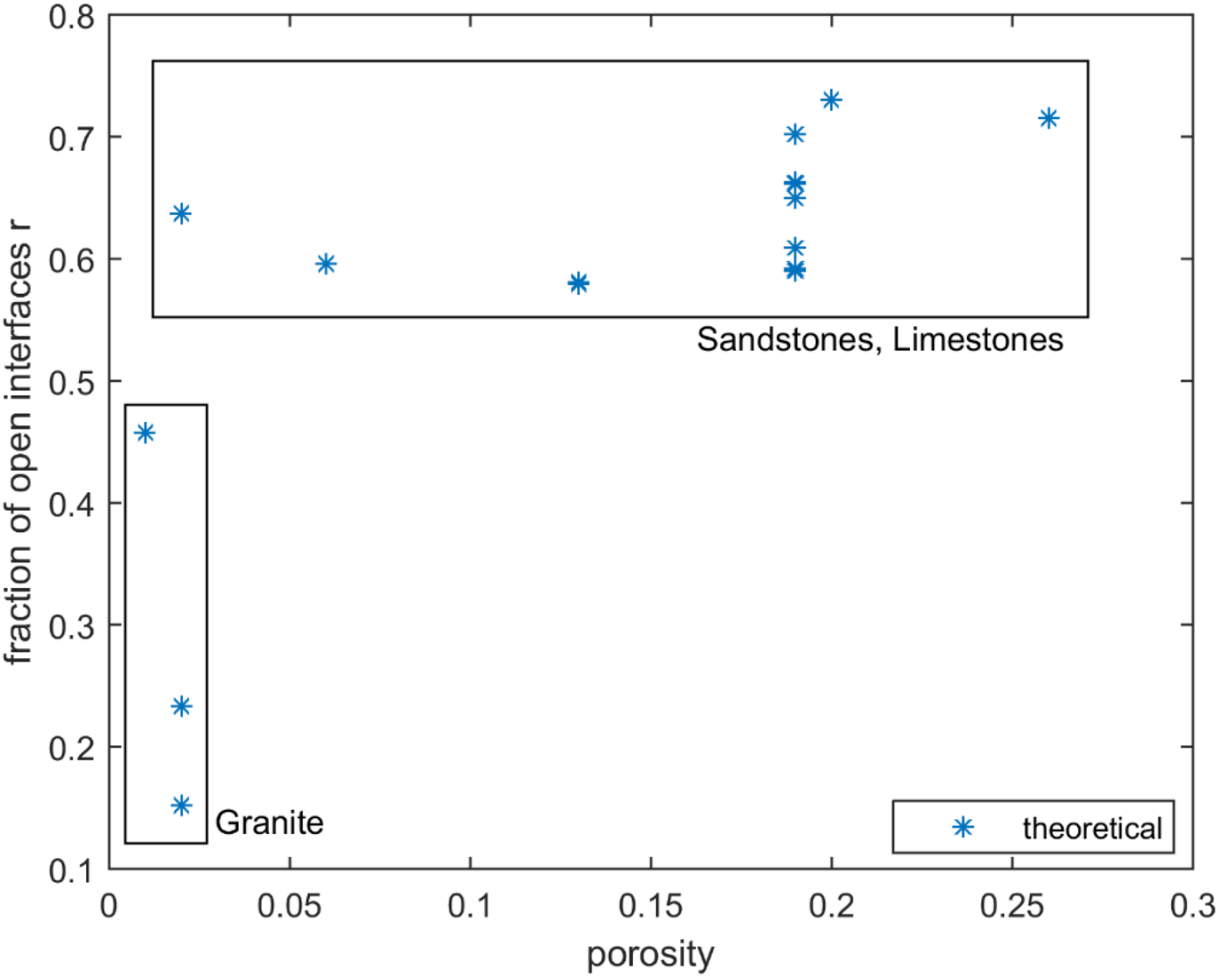
Fraction of open interfaces $r$ versus porosity. [The limestones data points are easy to identify by the porosity value of 0.13. Keeping two types of rocks (sandstones and limestones) in one group seems possible in this case because they have similar values for fraction of open interfaces].

Figure 5 shows estimates for the parameter $r$ for rocks given in Tables 1 and 2. The parameter $r$ is plotted as a function of the porosity $\phi$ and the value of $r$ is obtained from (12) by matching the Biot coefficient $\alpha$ with the experimental values, indicated in Tables 1 and 2. The grains are modelled as nearly spherical shapes and the pore space is also modelled as a nearly spherical shape. The results can be influenced by the shape of the pores and this was addressed in a previous study^53^.

**Figure 6:**
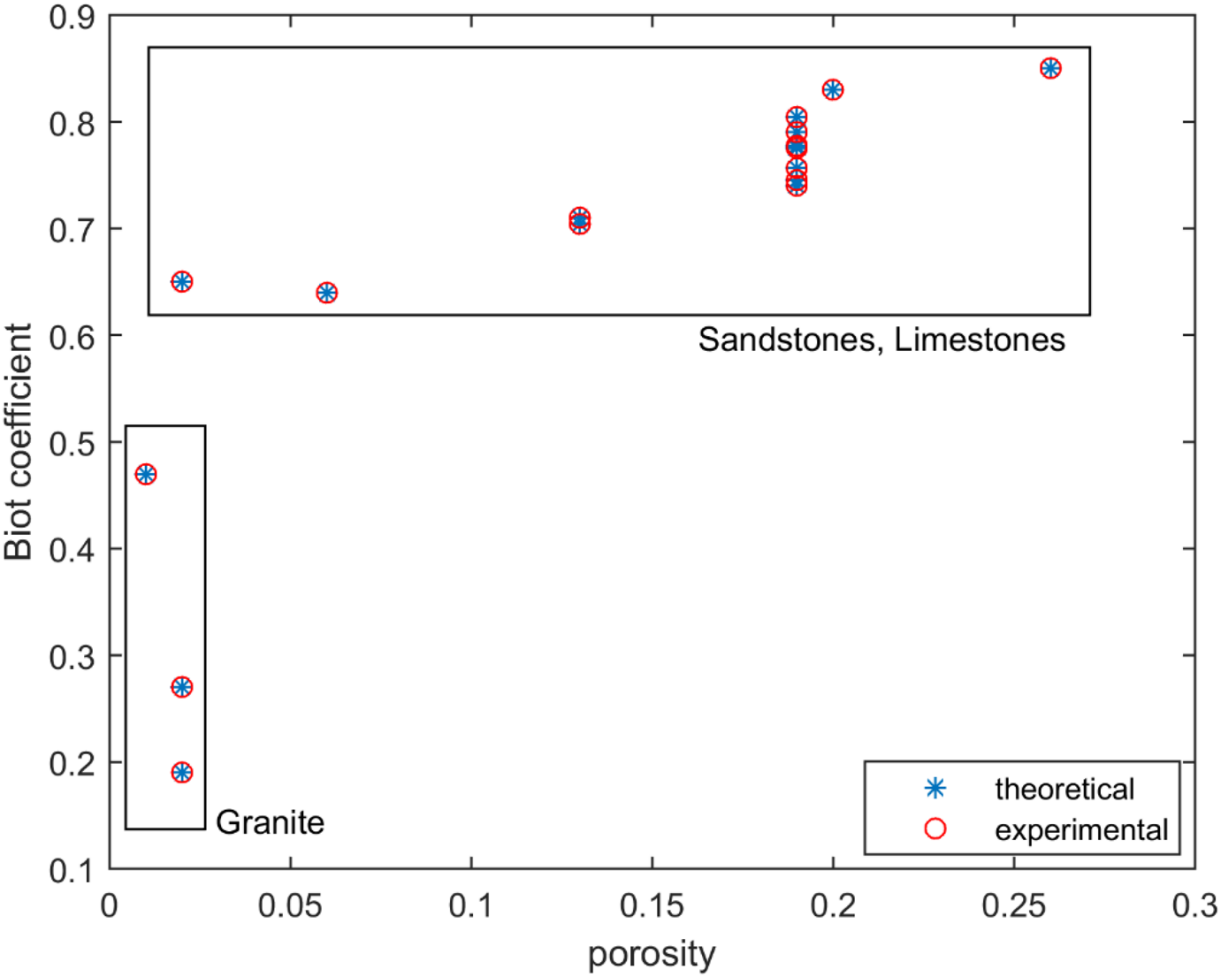
Biot coefficient $\alpha$ versus porosity.

In Fig. 6 we indicate the dependency of the Biot coefficient $\alpha$ on the porosity $\phi$. This figure demonstrates that by properly choosing the microstructural parameters $M$ (or $K$ ), $\rho$ and $r$ it is possible to exactly match the elastic constants $K^{eff}$, $G^{eff}$ and $\alpha$ with the experimental data. It can be observed from Fig. 6 that, on average, for sandstones and limestones the Biot coefficient is equal to 0.75 and for the marbles-granite group the Biot coefficient can be lower and in the range 0.2 to 0.5. This observation is consistent with results obtained recently for the Lac du Bonnet granite recovered from the Canadian Shield^79^.

The self-consistent estimate of Biot modulus $N$ can be obtained from the relationship^57^

13 $$\frac{1}{N} = \frac{3\alpha }{2}(1 - r)\left( {\frac{{1 - \frac{\phi }{{\left\{ {1 - \frac{3K}{2}} \right\}}}}}{{K_{n} R}}} \right) = \frac{3\alpha }{2}(1 - r)\left( {\frac{{1 - \phi \left( {\frac{1 - r}{{\alpha - r}}} \right)}}{{K_{n} R}}} \right)$$

When the estimates for the Biot modulus $N$ obtained from the relationship (13) are compared with the experimental values of $N$ there is a large discrepancy. To eliminate this discrepancy, the compressibility of the fluid phase must be taken into account, which gives another expression for the Biot modulus

14 $$\frac{1}{N} = \frac{\phi }{{K_{f} }} + \frac{3\alpha }{2}(1 - r)\left( {\frac{{1 - \phi \left( {\frac{1 - r}{{\alpha - r}}} \right)}}{{K_{n} R}}} \right)$$

where $K_{f}$ is the bulk modulus of the fluid. The interface stiffness ${K}_{n}R$ was estimated first from Eq. (9). This value is then substituted into (13) or (14) to estimate the Biot modulus $N$. Thus, a knowledge of the grain size radius is not required.

**Figure 7:**
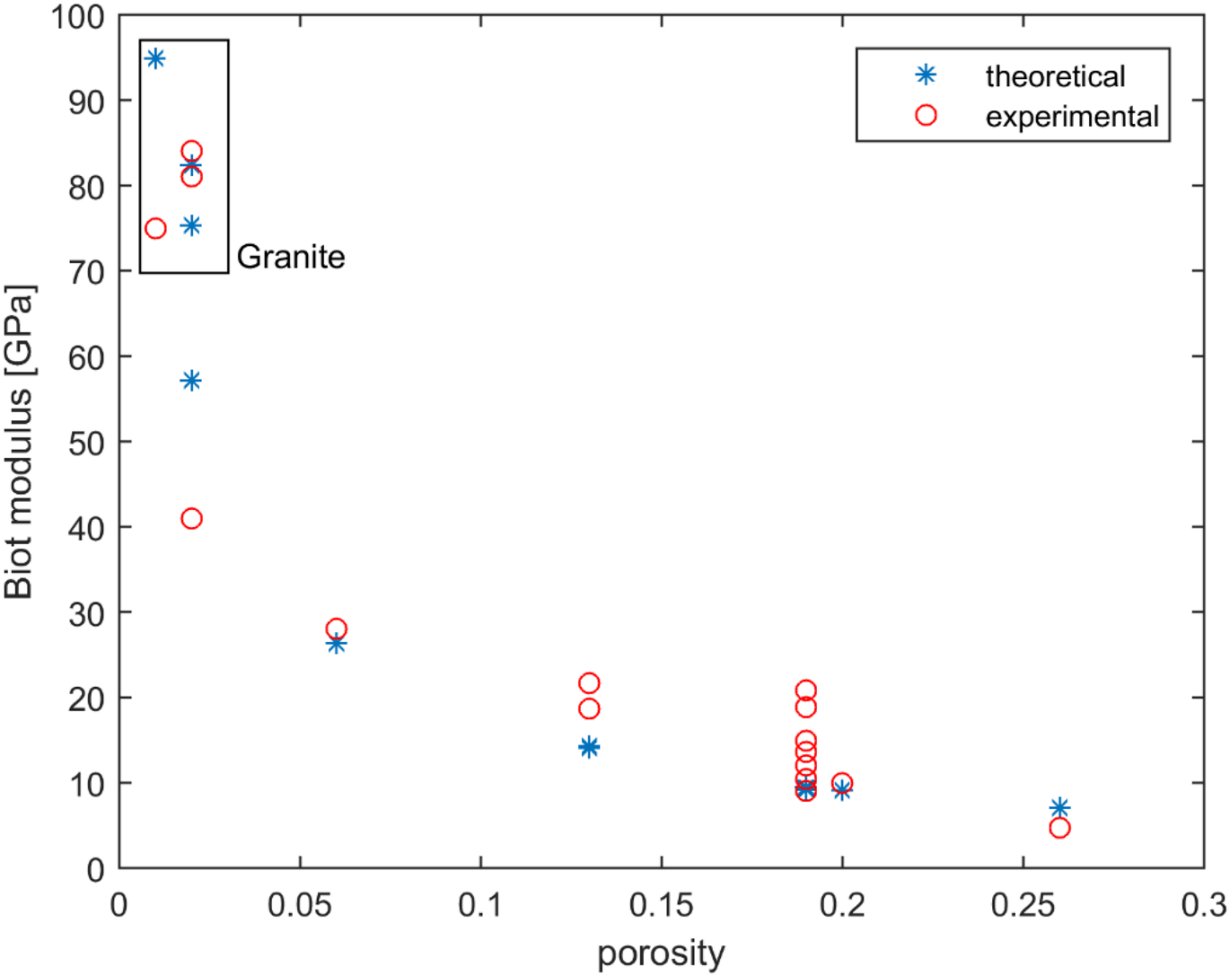
Biot modulus $N$ versus porosity.

Figure 7 shows experimental values of the Biot modulus $N$ and the corresponding theoretical estimates for $N$ obtained from (14). Theoretical values are obtained by setting the bulk modulus of the fluid $K_{f} = 2\;{\text{GPa}}$, which is typically applicable to water. An acceptable agreement between the theoretical and experimental values is obtained by choosing this constant value of $K_{f}$.

## Estimates for permeability

The self-consistent estimate of permeability $k$ is obtained as

15 $$k = \frac{2}{1 - 3\phi }\frac{r\eta }{R}$$

where $\eta$ is the interface permeability, which can be related to the aperture of the contact through the usual parallel plate model^55,80–83^. i.e.

16 $$\eta = \frac{{e^{3} }}{24}$$

Here $e$ is the spacing between two neighboring grains (joint opening) and $R$ is the grain radius. By substituting (16) into (15) we obtain

17 $$k = \frac{r}{12(1 - 3\phi )}\frac{{e^{3} }}{R}$$

The result (17) contains the additional factors that can influence permeability, including the grain size, bulk porosity and a measure of the fraction of open interfaces. If these parameters can be accurately estimated, the result gives an added perspective for estimating permeability. Since the equation for permeability (17) is used as an equality constraint in the optimization problem and the solution to the optimization problem exists, then the proper choice of grain radius and joint opening allows us to match the permeability obtained from theoretical estimates (17) with the experimental data.

It should be noted that the definition of the hydraulic aperture does not account for any fine particles that can be present in sandstone and carbonate rocks. As has been suggested^84^, an average value for the joint opening can be set as $e = 0.5\;{\mu m}$ and for the radius $R = 50\;{\mu m}$. It should be noted that these assigned values are typical of tight sandstones and should not be construed as an “average value”. They fulfil the function of normalizing values. However, by using these particular values it is not possible to match experimental values of permeability, presented in Table 1, with the theoretical estimate (17). Thus, we can introduce the following quantities, which represent deviations from established values

18 $$R_{n} = \frac{R}{{50\;{\mu m}}}\quad ;\quad e_{n} = \frac{e}{{0.5\;{\mu m}}}$$

When the results (18) are substituted into (17), we obtain

19 $$k = \frac{r}{12(1 - 3\phi )}\frac{{e_{n}^{3} (0.5)^{3} }}{{50R_{n} }} \times 10^{ - 12}$$

We can rewrite (19) as an equality constraint in terms of variables $R_{n}$ and $e_{n}^{3}$: i.e.

20 $$\frac{600k(1 - 3\phi )}{{r(0.5)^{3} }}10^{12} R_{n} - e_{n}^{3} = 0$$

Here $k$ is the value of permeability measured experimentally. As the objective function to be minimized, we can take the sum of deviations of variables $R_{n}$ and $e_{n}$ from unity i.e.,

21 $$\min :S = R_{n} ({\text{if}}\;R_{n} \ge 1) + \frac{1}{{R_{n} }}({\text{if}}\;R_{n} < 1) + e_{n} ({\text{if}}\;e_{n} \ge 1) + \frac{1}{{e_{n} }}({\text{if}}\;e_{n} < 1)$$

We note that $R_{n}$ and $e_{n}^{3}$ must be positive, which constitutes inequality constraints.

Solution to this non-linear optimization problem for the rocks presented in Table 1 is shown in Table 3. The solution was obtained with the help of MATLAB function fmincon. It may be observed that $R_{n}$ and $e_{n}$ are close to unity only for Berea sandstone and Boise sandstone. For other sandstones, the radius $R$ can be twice $50\;{\mu m}$ and the spacing between neighboring grains can be 5–7 times smaller than the normative value of $0.5\;{\mu m}$. The largest deviations are observed for the granite-marble group. Here, the grain radius $R$ is about 5 to 6 times larger than the normative value of $50\;{\mu m}$, and $e$ is , on average, 20 times smaller than $0.5\;{\mu m}$.

**Table 3:** Deviations of radius of the grain *R* and joint opening *e* from their nominal values of 50 µm, and 0.50 µm, respectively.

Rock	$R_{n}$	$e_{n}$
Ruhr sandstone	2.269007	0.146912
Tennessee marble	5.960993	0.055916
Charcoal granite	6.638004	0.050212
Berea sandstone	1.000000	0.836472
Westerly granite	5.510982	0.060483
Weber sandstone	1.544066	0.215891
Ohio sandstone	1.176403	0.283350
Pecos sandstone	2.055110	0.162203
Boise sandstone	1.000000	1.052523

## Concluding remarks

The estimation of the poroelasticity properties of rocks can proceed along two avenues; the first involves their direct evaluation from experimental data and the second approach is to utilize the theories developed for multiphasic composites to arrive at estimates for the poroelasticity parameters. Both approaches have their advantages and disadvantages. The purely experimental approach is void of consideration of the micro-mechanical input to the poroelastic parameters but provides the data set that can be used in the engineering calculation of poroelastic responses. The approaches based on multiphasic theories have to rely on idealized theoretical assumptions that lead to parameter estimates but adds a new dimension by introducing the properties of the fabric that compose the poroelastic material. The prudent option is to rely on both approaches and to take advantage of the merits of each approach to provide the validations that will enable the assignment of poroelastic parameter estimates. The results of the paper provide correlations between (1) porosity of a poroelastic solid and the grain-grain contact stiffnesses at the particulate level, (2) the interface stiffness ratio and effective shear to bulk modulus ratio, (3) open interface fraction and porosity, (4) the variation of the Biot coefficient and Biot modulus on porosity and (5) the dependency of permeability on microstructural properties. The research utilizes the material data available in the literature and provides the summary related to (1) to (4) in terms of the basic rock types relevant to sedimentary (sandstones, limestones) and igneous (granite, basalt) rocks. The data presented enables the preliminary identification of poroelastic parameters, which can be complemented by a rigorous program of laboratory tests. In the absence of experimental data for poroelastic properties of a particular rock, the findings of the research can be treated as a data set for preliminary geosciences calculations that requires recourse to the theory of linear poroelasticity. Since the original experimental data does not contain ranges or error estimates, the study can provide only firm values of the parameters. The measurement of the properties of saturated rocks with low permeability is generally a challenging task in the laboratory since it is difficult to ensure that the sample is fully saturated during testing. Such theoretical approaches can have practical applications in geotechnical engineering and rock mechanics. Finally, the paper presents a canonical methodology for including additional parameters in the development of concepts for examining poroelastic parameters. In efforts of this nature, the microstructural parameters are introduced within the framework of a plausible concept. The progress of the modelling will require novel experimental methodologies, in the area of geomechanics and material science for accurately estimating the parameters arising from the developments.
